# Editorial: Mental health stigma and UN Sustainable Development Goals

**DOI:** 10.3389/fpsyt.2023.1190406

**Published:** 2023-04-05

**Authors:** John Goodwin, Umer Zaman

**Affiliations:** ^1^University College Cork, Cork, Ireland; ^2^Endicott College of International Studies (ECIS), Woosong University, Daejeon, Republic of Korea

**Keywords:** United Nations, Sustainable Development Goals, mental health, stigma, wellbeing

Mental health has long been declared as human right (1), and, as noted by the World Health Organisation (2), there can be no health or sustainable development without mental health. In relation to sustainable development, the United Nations (3), under the 2030 Agenda for Sustainable Development, outline 17 Sustainable Development Goals (SDGs), which provide a blueprint for United Nations Member States working in partnership to improve health, education, economic growth, and reduce inequality, in addition to tackling climate change (1, 3).

Mental health is central to SDG 3, which is focused on good health and wellbeing, but it is also connected to other SDGs, owing to the relationship between mental health and poverty (SDG 1) and the reduction of inequalities (SDG 10), in addition to other SDGs. Therefore, addressing UN Sustainable Development Goals is hardly achievable without giving sufficient attention to mental health (2, 3).

However, there is evidence that much more work is needed with regards to the SDGs in promoting societal mental health and wellbeing. According to the World Health Organisation (4), only 49 countries, or 25% of World Health Organisation member states, have effectively integrated mental health into primary health care systems. Consequently, comprehensive mental health care may be out of reach for a significant proportion of the global population. Moreover, a large proportion of the population demonstrate poor help-seeking behaviors owing to mental health stigma (5–7), eroding the benefits of early intervention and leading to negative mental health outcomes. In comparison to physical health, the global focus on mental health is highly deficient in terms of budgets, education, and practice (4, 8).

The scope of this issue of Frontiers in Psychiatry was open to any work related to global mental health (and, more specifically, global mental health stigma) within the framework of the UN Sustainable Development Goals (3, 4). The topic has a particular interest in generating links between the various policy interventions to promote mental health and prevent mental health stigma (1, 3, 8).

Each paper in this Research Topic addresses SDG 1 (good health and wellbeing). Three studies address SDG 4 (quality education): Meng explore the relationship between mental health education and psychological wellbeing, in the context of the moderating role of politically motivated internet addiction and the ideological passion of college students. Gao investigates the relationships between project-based learning and mental health awareness with effective English language teaching. Pei explores the psychological factors associated with the academic performance of Chinese-English bilinguals as an outcome of their emotional competence, flipped learning readiness, and mental health stigma. Lim et al., in their meta-analysis, focus on SDG 16 (peace, justice and strong institutions), investigating the aggregate prevalence of depression, anxiety and post-traumatic stress symptoms among both civilian and military populations exposed to war. Qiu et al. explore the connection between mindfulness and sustainable economic behavior, and the mediating role of emotional labor and the mental health of farmers in China, thus addressing SDG 12 (responsible consumption and production).

## Author contributions

Both authors listed have made a substantial, direct, and intellectual contribution to the work and approved it for publication.

## References

[1] United Nations General Assembly. Universal Declaration of Human Rights (Vol. 3381). Department of State, United States of America (1949).

[2] World Health Organisation (2022). Mental Health. Geneva: World Health Organisation. Available online at: https://www.who.int/news-room/fact-sheets/detail/mental-health-strengthening-our-response (accessed February 20, 2023).

[3] United Nations. Transforming our World: The 2030 Agenda for Sustainable Development. New York, NY: UN General Assembly (2015).

[4] World Health Organisation. Mental Health Atlas 2020. Geneva: World Health Organisation (2020).

[5] Doll CM Michel C Rosen M Osman N Schimmelmann BG Schultze-Lutter F. Predictors of help-seeking behaviour in people with mental health problems: a 3- year prospective community study. BMC Psychiatry. (2021) 21:432. 10.1186/s12888-021-03435-4 34479537 PMC8414662

[6] Goodwin J Behan L Kelly P McCarthy K Horgan A. Help-seeking behaviours and mental wellbeing of first year undergraduate University students. Psychiatry Res. (2016) 246:129–35. 10.1016/j.psychres.2016.09.015 27693865

[7] Shafie S Subramaniam M Abdin E Vaingankar JA Sambasivam R Zhang Y. Help-seeking patterns among the general population in Singapore: results from the Singapore mental health study 2016. Admin Policy Mental Health Mental Health Serv Res. (2020) 48:586–96. 10.1007/s10488-020-01092-5 33057931 PMC8192323

[8] Mental Health Reform. Budget 2023. Mental Health Reform. (2023). Available online at: https://www.mentalhealthreform.ie/budget-2023/#:~:text=1.-,About%20Budget%202023,health%20services%20for%20Budget%202023 (accessed February 20, 2023).

